# The Use of Pleural Adenosine Deaminase in the Early Diagnosis and Treatment of Spinal Tuberculosis

**DOI:** 10.7759/cureus.23668

**Published:** 2022-03-30

**Authors:** Mathew C Finniss, Paul Lewis, Paras Patel

**Affiliations:** 1 Department of Infectious Diseases, East Tennessee State University Quillen College of Medicine, Johnson City, USA; 2 Pharmacy, Johnson City Medical Center, Johnson City, USA; 3 Infectious Diseases, Karius Inc., Redwood City, USA

**Keywords:** adenosine deaminase, tuberculous pleural effusion, afb culture, afb smear, tuberculosis spondylitis

## Abstract

Spinal tuberculosis (TB) is associated with serious neurologic morbidity. It commonly presents as back pain, with or without systemic symptoms. Magnetic resonance imaging (MRI) is the most sensitive and specific imaging modality for spinal TB. The diagnosis of spinal TB is made with tissue biopsy and acid-fast bacilli (AFB) culture; however, tissue AFB smear and tissue TB deoxyribonucleic acid (DNA) polymerase chain reaction (PCR) can influence early clinical decision making. Ancillary tests such as the purified protein derivative (PPD) skin test, QuantiFERON®-TB Gold (QFT) or pleural adenosine deaminase (ADA) can be used in conjunction with radiology and clinical findings to initiate treatment while AFB tissue cultures are pending. Spinal TB responds well to early medical management and surgery is reserved for cases with neurologic complications.

## Introduction

Spinal tuberculosis (TB) is important to recognize early and treat aggressively. Tissue acid-fast bacilli (AFB) culture is the preferred diagnostic test; however, cultures can take more than six weeks and tissue samples can be difficult to obtain [1]. Treatment should not be delayed for culture results. Tissue AFB smear and tissue TB DNA polymerase chain reaction (PCR), which are more rapid than tissue AFB culture, can be used to guide early therapy, however, up to 50% of tissue AFB smears in spinal TB are negative and TB DNA PCR for spinal TB is poorly sensitive [1].

The diagnosis and treatment of spinal TB can be challenging in the setting of negative tissue AFB smear, negative tissue TB DNA PCR and pending tissue AFB culture. In these cases, treatment may be initiated based on radiology and clinical findings alone [1]. Ancillary tests such as purified protein derivative (PPD) and QuantiFERON®-TB Gold (QFT; Qiagen, Germantown, MD, USA) have varying sensitivities and specificities for spinal TB but can also be used to help guide early decision making [1,2]. The sensitivity and specificity of pleural adenosine deaminase (ADA) in spinal TB are unknown.

Herein, we report a case of spinal TB in which characteristic MRI findings and pleural ADA were used to initiate spinal TB treatment. In our case, tissue AFB cultures required 20 days, which is an unacceptably long time for a morbid disease that requires early treatment. In the setting of negative tissue AFB smear, negative tissue TB DNA PCR and pending AFB culture, pleural ADA can be used to direct early clinical decisions and reduce neurologic complications.

## Case presentation

**Figure 1:**
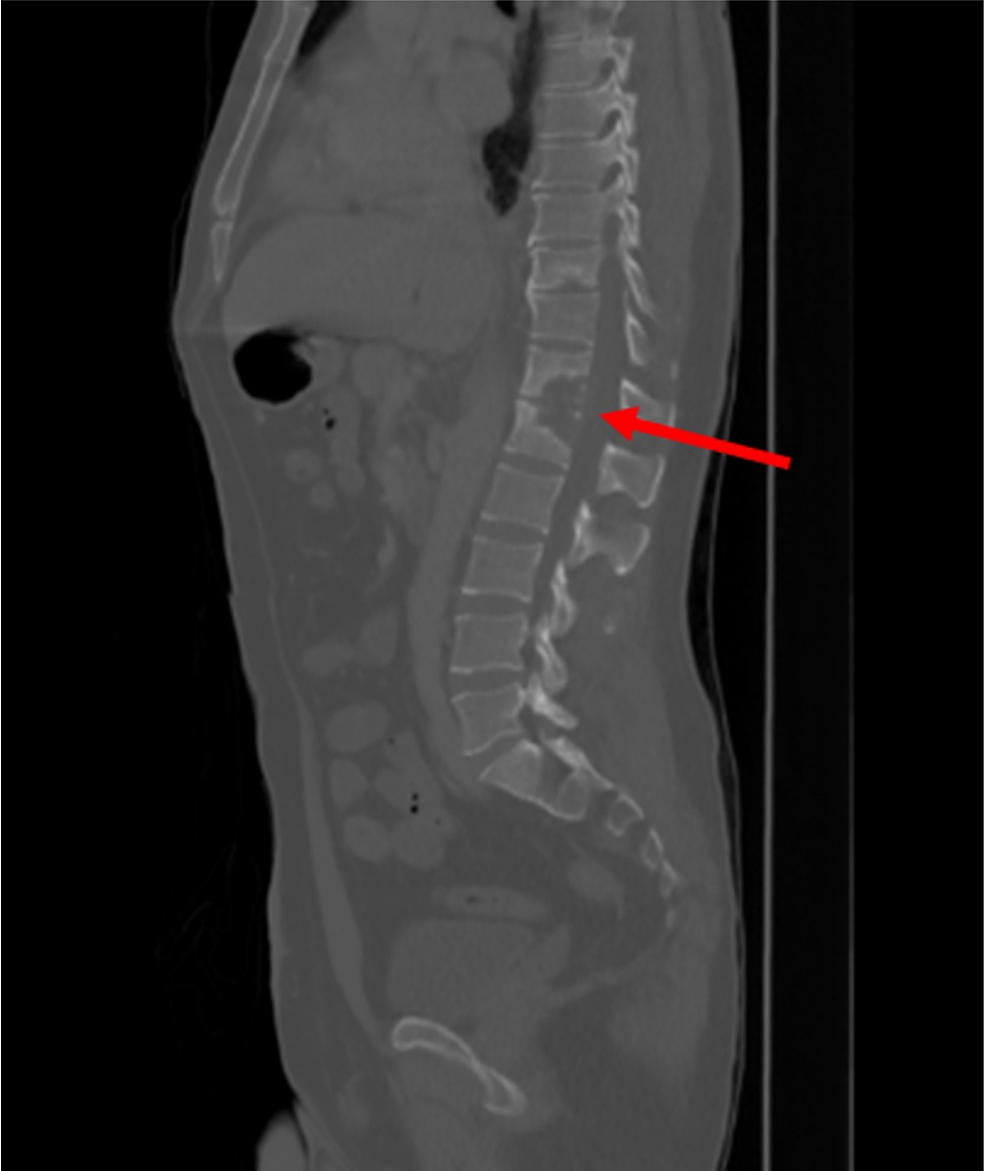
CT scan demonstrating destruction of T12 - L1 vertebral bodies (red arrow)

A 38-year-old Hispanic male presented with fever and back pain. On admission he had a temperature of 101.9F, a heart rate of 93 beats per minute (BPM) and a blood pressure of 151/110 mmHg. The physical exam was significant for low back tenderness with palpation. A complete blood count (CBC) and basic metabolic panel (BMP) were unremarkable. An abdominal CT demonstrated compression deformities of the T12 - L1 vertebrae (Figure 1).

Blood cultures were collected and were negative. Empiric vancomycin and ceftriaxone were started. Biopsy of the T12 - L1 vertebrae was done. Bacterial and fungal tissue cultures were negative. Tissue AFB smear was negative. Tissue AFB culture was sent. A PPD skin test was placed and a QFT was collected.

A CT chest demonstrated a right pleural effusion, and a diagnostic thoracentesis was performed. The pleural protein was 5.3 g/dL, pleural lactate dehydrogenase (LDH) was 669 U/L and pleural ADA was 26 IU/L. AFB smear and TB DNA PCR (MTB complex PCR) of the pleural fluid were negative. Bacterial and fungal cultures of the pleural fluid were negative. Pleural fluid AFB culture was sent. Vancomycin and ceftriaxone were discontinued. Rifampin, isoniazid, pyrazinamide, and ethambutol were started. The PPD skin test and QFT returned positive. Sputum AFB smears were negative. A human immunodeficiency virus (HIV) 1/2 antibody screen was non-reactive.

The patient underwent repeat thoracentesis. AFB smear and TB DNA PCR of the pleural fluid were negative. Pleural fluid AFB culture was sent. The patient also underwent repeat T12 - L1 vertebral biopsy. Tissue AFB smear and tissue TB DNA PCR were negative. Tissue AFB culture was sent. The patient was discharged on rifampin, pyridoxine, pyrazinamide, isoniazid, and ethambutol. AFB culture from the initial thoracentesis and the repeat T12 - L1 vertebral biopsy returned positive after 28 and 20 days respectively for Mycobacterium tuberculosis. All other AFB cultures were negative.

## Discussion

The progression of spinal TB is insidious, and the presentation is non-specific. Back pain is the most reported symptom [2]. Constitutional symptoms occur in 20% - 30% of cases and are more common when pulmonary TB is also present [2]. The most common site of infection is the thoracolumbar junction [2]. Typically, contiguous vertebrae are involved, but 16% of cases also involve non-contiguous vertebrae [3]. Plain x-rays have poor sensitivity and specificity for spinal TB, however chest radiography is important, as 60% - 70% of spinal TB cases will also have pulmonary TB [2]. CT can detect anterior vertebral body destruction, vertebral collapse and disc space narrowing but MRI provides the best visualization of the neurologic structures [1,2].

Tissue AFB culture is highly specific and the preferred diagnostic test for spinal TB. Tissue samples, however, can be difficult to obtain, and tissue AFB cultures can take more than six weeks [1]. Tissue AFB smear and tissue TB DNA PCR can help guide early therapy while waiting for tissue AFB culture, however, 50% of tissue AFB smears are negative and tissue TB DNA PCR is poorly sensitive [1,2]. This creates a clinical dilemma in patients with negative tissue AFB smear and negative tissue TB DNA PCR with pending tissue AFB cultures. In these cases, ancillary tests such as PPD and QTF can also be used to initiate anti-TB therapy. The reported sensitivity of PPD and QTF in spinal TB is 40% - 55% and 50% - 65% respectively, while the reported specificity of PPD and QTF in spinal TB is 75% and 85% respectively [2]. When a concurrent pleural effusion is present pleural ADA can be used to establish the diagnosis of TB and initiate early treatment.

Pleural ADA has been studied as a rapid and cheap way to diagnose pulmonary TB. The interpretation of pleural ADA, however, can be challenging as it is plagued by false positives and false negatives, its predictive value depends on TB prevalence and there is no widely accepted diagnostic threshold of pleural ADA [4,5]. For example, smokers, elderly patients, and early TB infections are more likely to have false-negative pleural ADA while empyema, parapneumonic effusions, and malignant effusions are more likely to have false-positive pleural ADA [5]. In high TB prevalence areas, the positive predictive value (PPV) of pleural ADA is high, while in low prevalence areas, the negative predictive value (NPV) of pleural ADA is high [5].

Reported pleural ADA levels are typically between 40 IU/L - 60 IU/L in pulmonary TB, however, a few studies have reported good sensitivity and specify of pleural ADA levels < 40 IU/L in high prevalence areas [6,7]. Verma et al. reported 100% sensitivity and 77% specificity for pleural ADA > 36 IU/L in 34 patients with pulmonary TB in Allahabad [6]. Likewise, Helmy et al. reported 80% sensitivity and 85% specificity for pleural ADA > 30 IU/L in 30 patients with pulmonary TB in Cairo. The PPV for pleural ADA > 30 IU/L was 83% [7]. The diagnostic utility of pleural ADA can be increased by measuring the ADA iso-enzymes or by calculating the pleural LDH/ADA ratio [8]. Measurement of pleural ADA-2 isoenzyme increases the specificity for TB pleural effusions by 5% [9]. An LDH/ADA ratio < 16.2 in high TB prevalence areas is 93% sensitive and 93% specific for TB pleural effusion while an LDH/ADA < 15 is 89% sensitive and 85% specific for TB pleural effusions in low TB prevalence areas [10]. The sensitivity and specificity of pleural ADA in spinal TB are not known.

In our report, pleural ADA was used to secure the diagnosis of TB and initiate anti-TB therapy in a case of spinal TB with negative tissue AFB smear, negative tissue TB DNA PCR and pending tissue AFB culture. Although the pleural ADA was not greater than the widely accepted threshold of 40 IU/L for pulmonary TB, several studies have demonstrated reasonable sensitivity and specificity for lower thresholds [6,7]. Furthermore, our patient was a smoker which may have contributed to a false negative pleural ADA. Due to the propensity for false positives and false negatives, pleural ADA should be interpreted in the overall clinical context rather than compared against a diagnostic threshold. AFB culture from the repeat T12 - L1 vertebral biopsy required 20 days, which is too long to delay the treatment of a seriously morbid disease.

## Conclusions

Spinal TB can lead to serious neurologic morbidity; therefore, treatment should be initiated as soon as possible. Tissue AFB culture is the preferred diagnostic test; however, it can take more than six weeks and delay clinical decision making. Characteristic imaging findings as well as tissue AFB smear and tissue TB DNA PCR can be used to guide early clinical decisions. Ancillary tests such as PPD, QFT and pleural ADA can be used to secure the diagnosis of TB and guide early clinical decisions especially when the tissue AFB smear and the tissue TB DNA PCR are negative. Our case highlights the use of pleural ADA to initiate early treatment in a case of spinal TB with negative tissue AFB smear and pending AFB cultures. The main disadvantages of pleural ADA in the diagnosis of spinal TB are that it cannot be used for the 30% - 40% of patients without concurrent pulmonary TB and its interpretation can be challenging.
